# Comparison of facial soft tissue thickness, pharyngeal airway widths in different skeletal Patterns

**DOI:** 10.1590/2177-6709.30.3.e2524217.oar

**Published:** 2025-10-13

**Authors:** Chraseeta LOUIS, Siddharth RAGHAVA, Praveena SHETTY, George S. MANDOLIL, Ajay Rai E-ELANTHAJE, Beniya E. S-ELANJIKKAL SILVESTER

**Affiliations:** 1 Srinivas Institute of Dental Sciences, Dental School (Mangalore, India).; 2 Srinivas Institute of Dental Sciences, Dental School, Department of Orthodontics and Dentofacial Orhopaedics (Mangalore, India).; 3 Annoor Dental College & Hospital, Dental School (Kochi, India).

**Keywords:** Facial profile, Soft tissue thickness, Airway, Skeletal pattern, Cephalometrics, Perfil facial, Espessura do tecido mole, Vias aéreas, Padrão esquelético, Cefalometria

## Abstract

**Methods:**

The sample was divided into skeletal Patterns I, II and III, based on ANB and Wits appraisal: Pattern I, when ANB angle = 0-4° and BO was ahead AO by 1mm in males (SD = 1.17 ± 1.9mm), 0mm in females (SD = -0.10 ± 1.77mm); Pattern II, when ANB >4° and BO was located behind AO (43 subjects); and Pattern III, ANB < 0° and BO was ahead of AO (43 subjects). Each group had 43 lateral cephalograms, with a total of 129 cephalograms. The samples were divided into three groups only if they satisfied the criteria of both ANB and Wits appraisal. For this, 26 selected landmarks were identified and, using 13 linear measurements, facial soft tissue thickness and pharyngeal airway widths were measured and compared among the different skeletal patterns.

**Results:**

There was statistically significant difference in facial soft tissue thickness in the different skeletal patterns. *Labrale superius* and stomion were statistically significant and higher in Pattern III and smaller in Pattern II. *Labrale inferius* was found to be statistically significant and higher in Pattern II and smaller in Pattern III. Though not statistically significant, the thickness at Menton, Pogonion, and Gnathion was higher in Pattern II, average in Pattern I and smaller in Pattern III. Sexual dimorphism was noted: Men had a greater soft tissue thickness than women in all sites. There was statistically significant difference in pharyngeal airway widths in different skeletal malocclusions. Both upper and lower pharyngeal airway widths were found to be statistically significant and higher in Pattern III and smaller in skeletal Pattern II.

**Conclusion:**

When developing orthodontic treatment plans, soft tissue differences and pharyngeal airway widths need to be taken into account as crucial factors. The findings of the current study indicate that there was a substantial correlation between the thickness of the soft tissues in the face, the diameter of the pharynx, and several skeletal patterns.

## RESUMO

**Métodos:**

A amostra foi dividida em Padrões esqueléticos I, II e III, com base no ANB e na avaliação de Wits: Padrão I, ângulo ANB = 0-4° e BO à frente do AO em 1 mm nos homens (DP = 1,17 ± 1,9 mm) e 0 mm nas mulheres (DP = -0,10 ± 1,77 mm); Padrão II, ANB >4° e BO localizado atrás do AO (43 indivíduos); e Padrão III, ANB < 0° e BO à frente do AO (43 indivíduos). Cada grupo teve 43 cefalogramas laterais, com um total de 129 cefalogramas. As amostras foram incluídas nos grupos somente se satisfizessem os critérios de ANB e avaliação de Wits. Para isso, 26 pontos de referência selecionados foram identificados e, usando 13 medidas lineares, a espessura dos tecidos moles faciais e a largura das vias aéreas faríngeas foram medidas e comparadas entre os diferentes padrões esqueléticos.

**Resultados:**

Houve diferença estatisticamente significativa na espessura dos tecidos moles faciais nos diferentes padrões esqueléticos. O *Labrale superius* e o Estômio foram significativamente maiores no Padrão III e menores no Padrão II. O *Labrale inferius* foi significativamente maior no Padrão II e menor no Padrão III. Embora não estatisticamente significativa, a espessura no Mento, Pogônio e Gnátio foi maior no Padrão II, média no Padrão I e menor no Padrão III. Foi observado dimorfismo sexual: os homens apresentaram maior espessura de tecidos moles do que as mulheres, em todos os locais. Houve diferença estatisticamente significativa nas larguras das vias aéreas faríngeas nos diferentes padrões esqueléticos. As larguras das vias aéreas superiores e inferiores da faringe foram significativamente maiores no Padrão III e menores no Padrão II.

**Conclusão:**

Ao desenvolver planos de tratamento ortodôntico, as diferenças dos tecidos moles e as larguras das vias aéreas faríngeas precisam ser levadas em conta como fatores cruciais. Os resultados do presente estudo indicam que houve uma correlação substancial entre a espessura dos tecidos moles da face, o diâmetro da faringe e os diferentes padrões esqueléticos.

## INTRODUCTION

The human face is the most important and characteristic part of the human body.^1^

As human beings, our emotions get reflected in our face^1^. It is not just the beauty that contributes to an attractive face, but also the health, the wisdom, social accomplishments, internal happiness etc.^1^ Facial soft tissue thickness (FSTTs), dental and skeletal characteristics are some of the factors that determine the profile of an individual.^2^

Muscles, subcutaneous fat, soft tissue, and skin can develop proportionately or disproportionately, corresponding to underlying skeletal structures.^1^ Hence, we should consider both hard and soft tissue norms for establishing harmonious facial aesthetics and an optimal functional occlusion.^3^

Often severe skeletal discrepancy is masked by favorable soft tissue. Nature has a tendency of compensation, be it hard tissues for the soft tissues or vice versa.^4^ Increased soft tissue thickness was reported where there is anteroposterior skeletal jaw deficiency.^5^ ^,^ ^6^ Hence the soft tissue thickness of every patient is an important factor to consider during orthodontic assessment.

Pharyngeal airway space (PAS) is the soft tissue space that is bounded superiorly by nasopharynx, inferiorly by epiglottis, anteriorly by maxillomandibular complex, and posteriorly by spinal column.^7^ Any of the dentomaxillofacial deformities like retrusive maxilla or mandible can cause changes in the volume of PAS, as hence affect breathing.^8^ ^-^ ^10^

Facial soft tissue plays a crucial role in facial aesthetics.^11^ Facial aesthetics is an important goal of treatment for contemporary orthodontics and it is one of the patient’s main reasons for seeking orthodontic treatment. Thus, the assessment of the patient’s facial soft tissue is very important for orthodontic diagnosis and treatment planning. Currently, there is paucity of evidence exploring the influence of the soft tissue thickness and pharyngeal airway widths in different skeletal malocclusions that help in determining the therapeutic strategy. 

Considering this context, the present study was designed to measure FSTTs and pharyngeal airway widths of adult individuals, to investigate the influence of soft tissues and soft tissue compensations as a causative factor for different skeletal patterns. 

The data from this study can aid the clinician for planning of the appropriate treatment plan, as treatment should be planned not just based on different skeletal patterns, but also considering the soft tissue coverage over skeletal base.

## MATERIAL AND METHODS

Lateral cephalograms were collected from the Department of Oral Medicine and Radiology (Srinivas Institute Of Dental Science, India), and classified into Pattern I, Pattern II, Pattern III, based on ANB^11^ and Wits appraisal:^12^ Pattern I, ANB = 0-4°, and BO ahead of AO by 1mm in males (SD = 1.17±1.9mm), and 0mm in females (SD = -0.10±1.77mm) (43 subjects); Pattern II, ANB > 4°, and BO located behind AO (43 subjects); and Pattern III, ANB < 0°, and BO ahead of AO (43 subjects). The samples were divided into three groups only if they satisfied the criteria of both ANB and Wits appraisal.

These 129 digital cephalograms were transferred to computer imported to Facad^®^ cephalometric tracing software (version 3.11, Ilexis AB Linkoping, Sweden version), and 22 selected landmarks were identified and, using 11 linear measurements, facial soft tissue thickness were measured (Fig. 3) and compared among the different skeletal patterns. Four selected landmarks were identified and, using two linear measurements, pharyngeal airway width was measured among the different skeletal malocclusions (Fig. 4). McNamara analysis^13^ was chosen for airway measurement, and variables were generated automatically.

### INCLUSION CRITERIA

- Age between 16 to 30 years: subjects selected in this study were of the age group 16-31 years, where no much changes occur, and the soft tissue thickness already have reached adult size, according to Subtenly.^18^
- Patients from both sexes were included.
- No history of previous orthodontic treatment, plastic surgery or orthognathic surgery.

### EXCLUSION CRITERIA

- Individuals with craniofacial syndromes, facial scars, evident facial asymmetry, cleft lip and palate and with a major illness.
- Individuals with pharyngeal pathology or nasal obstruction.
- Individuals with history of trauma, fall, surgery of nose.
- Patients who had undergone previous surgeries for pharyngeal pathologies.

## METHODS

- Out of the thirteen linear measurements used, eleven measures the soft tissue thickness for different skeletal patterns, and the remaining two measures the upper and lower airways widths, as in McNamara analysis.
- The thirteen linear measurements used are described in Table 1, with a brief description.

**Table 1:** Description of the linear measurements used in the study.

MEASUREMENTS	DESCRIPTION
(1) Gls-GL	Distance from the most prominent point on the frontal bone to the soft tissue prominence on the forehead.
(2) Ns-N	Distance from the point nasion to the soft tissue nasion.
(3) Rh linear	Perpendicular distance from the intersection of the nasal bone and cartilage to the soft tissue.
(4) Sn-A	Distance between subnasale and A point.
(5) Ls-Pr	Distance between the most prominent point of the upper lip and prosthion.
(6) St-U1	Distance between the most prominent point of the upper incisor and stomion.
(7) Li-Id	Distance between the most prominent point of the lower lip and infradentale.
(8) B-Lm	Distance from point B to labiomental sulcus.
(9) Pogs-pog	The distance between soft tissue pogonion and bony pogonion.
(10) Mes-Me	The distance between soft tissue menton and bony menton.
(11) Gns-Gn	The distance between soft tissue gnathion bony and gnathion.
Upper airway width	Measured from the point on the posterior outline of the soft palate to the closest point on the distal wall of pharynx. Upper airway measures around 15-20 mm, whereas reduction by 2 mm or more indicates the impairment of the upper airway.
Lower airway width	Measured from the point of intersection of the distal portion of the tongue and the lower border of the mandible to the nearest point on the distal wall of pharynx, and it is approximately 11-14 mm, irrespective of the age.

**Figure 1:**
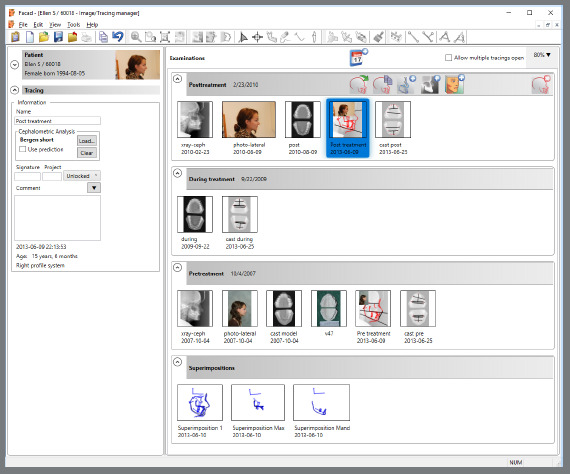
Patient registration.

**Figure 2:**
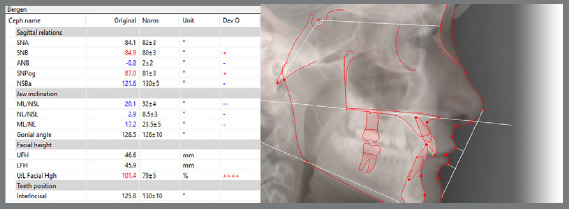
Analysis report, with tracing.

One hundred-twenty nine lateral cephalograms were imported to Facad^®^ software for analysis. After patient registration (Fig. 1) and calibration (the recommended minimum distance of calibration in Facad^®^ is at least 10 mm, with a minimum accuracy of ± 0.2 mm), landmarks were identified with the help of marker placement guide, which shows the correct marker position in the upper left corner of the image. The variables are generated automatically (Fig. 2), and measurements are entered into Excel spreadsheet (Microsoft, Seattle/WA).

A maximum of six radiographs were traced in one session, to prevent operator fatigue, and the same radiograph was not done immediately, to avoid the risk of memorization of landmarks. Reproducibility was tested after one month. Each analysis of same patient was done at different times, and the examiner was blinded of the previous measurements. Twenty-five percent of the cephalometric measurements were checked after four weeks interval, to assess the intraexaminer reliability.

The reliability was evaluated by using Pearson correlation coefficient. A t-test is used to assess the reproducibility. The significance level was set at p <0.05.

## STUDY DESIGN

**Figure 3:**
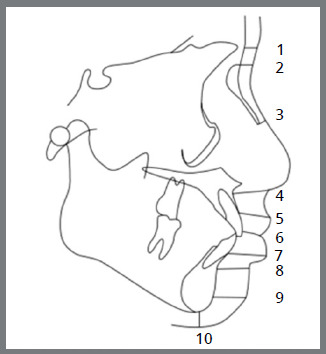
Linear measurements of facial soft tissue thickness: GLS-GL (1), NS-N (2), Rh linear (3), Sn-A (4), Ls-Pr (5), St-U1 (6), Li-Id (7), B-Lm (8), Pogs-pog (9), Mes-Me (10), Gns-Gn (11).

**Figure 4:**
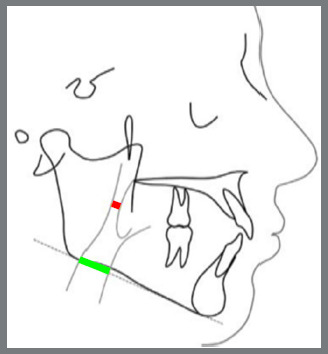
Landmarks for measuring upper and lower airway widths: red line = upper airway width, green line = lower airway width.

» Based on ANB angle and Wits appraisal, 43 lateral cephalograms were selected and divided into Pattern I, Pattern II, and Pattern III groups.
» Measurements were tabulated.
» The 129 digital cephalograms were imported to FACAD^®^ software.
» A total of 26 landmarks and 13 linear measurements were used to measure the FSTTs and pharyngeal airways widths: Gls-Gs, Ns-N, Rh linear, Sn-A, Ls-Pr, St-U1, Li-Id, B-Lm, Pogs-pog, Mes-Me, Gns-Gn, upper airway width, and lower airway width (Fig. 3 and 4).
» All analysis were performed by the same investigator and, after four weeks, measurements were rechecked for reliability using Pearson correlation coefficient, and for reproducibility using *t*-test.
» All values obtained were subjected to ANOVA test, to check whether the value obtained was statistically significant. Tukey test was performed after ANOVA, to check among which groups the clinical significance was present (Tables 2 to [Table t3] [Table t4] [Table t5] [Table t6] 7).

**Table 2:** Distribution and comparison of facial soft tissue thickness and pharyngeal airway widths in Pattern I, II and III skeletal malocclusion.

Malocclusion		Measurements												
		GLS-GL	NS-N	RH	SN-A	LS-PR	ST-U1	LI-ID	B-LM	POGS-POG	MES-ME	GNS-GN	Upper airway	Lower airway
Pattern I n=43	Mean	5.68	6.08	2.66^a^	15.38	14.85^a^	7.44 a	12.86^a^	12.50^a^	12.13	7.49	10.08	17.91^a^	13.29^a^
	Std. Deviation	0.93	1.73	0.93	2.85	2.6318	2.26	2.79	1.89	1.82	1.64	2.36	0.53	0.93
	Minimum	3.2	2.4	1.1	11.2	9.4	3.7	4.7	8.9	7.7	5.1	6.4	17.1	11.1
	Maximum	7.9	9.5	6.3	23.7	20.8	15.4	19.3	17.8	17.7	12.1	18.0	18.7	15.0
Pattern II n=43	Mean	5.49	6.34	2.84^a,b^	15.32	14.01^a,b^	7.59^a^	14.44^b^	12.54^a^	12.38	7.78	9.89	14.75^b^	10.99^b^
	Std. Deviation	0.87	1.92	0.91	2.01	2.0458	2.00	2.56	1.97	2.2402	1.69	2.75	0.39	0.40
	Minimum	3.8	2.3	1.1	11.6	10.5	3.8	10.6	8.6	8.0	4.9	5.5	14.2	9.7
	Maximum	7.7	10.0	5.3	19.7	18.6	11.5	18.9	17.7	17.7	12.3	16.0	15.7	11.7
Pattern III n=43	Mean	5.92	6.52	2.36^a^	15.87	12.90^b^	15.41^b^	9.41 c	14.08^b^	12.67	8.27	10.41	19.22^c^	14.77^c^
	Std. Deviation	0.96	1.83	0.63	3.02	2.80	2.10	4.34	2.70	2.26	2.66	2.52	0.67	.61
	Minimum	4.3	3.5	.9	11.2	8.4	11.2	4.6	9.0	8.7	4.4	6.9	18.1	13.2
	Maximum	8.8	10.1	3.0	21.0	18.1	18.8	17.4	17.1	17.1	13.3	16.9	20.5	15.8
F value		2.340	0.634	3.671	0.558	6.516	198.284	25.753	7.096	0.709	1.614	.461	783.621	335.318
p value		0.101	0.532	0.028*	0.574	0.002*	<0.001*	<0.001*	0.001*	0.496	0.203	0.632	< 0.001*	<0.001*

^a,b,c^ Different superscript letters along the columns indicate statistically significant differences determined using Tukey’s *post-hoc* test. F value from ANOVA. *p<.05 indicate statistical significance from ANOVA test.

**Table 3: t3:** Distribution and comparison of facial soft tissue thickness and pharyngeal airway widths in Pattern I, II and III skeletal malocclusion among female participants.

Malocclusion		Measurements												
		GLS-GL	NS-N	RH	SN-A	LS-PR	ST-U1	LI-ID	B-LM	POGS-POG	MES-ME	GNS-GN	Upper airway	Lower airway
Pattern I (n=26)	Mean	5.44^a^	5.67	2.53	14.35^a^	13.84	7.15a	12.47^a^	12.32^a^	11.86	6.79^a^	9.19^a^	17.89^a^	13.24^a^
	Std. Deviation	0.58	1.73	1.02	2.01	2.55	1.90	2.14	2.18	1.56	1.12	1.54	0.57	0.95
	Minimum	4.3	2.4	1.1	11.2	9.4	3.7	9.6	8.9	7.7	5.1	6.8	17.1	11.7
	Maximum	6.9	8.6	6.3	19.4	20.0	10.8	16.3	17.8	15.5	9.5	12.5	18.7	15.0
Pattern II (n=29)	Mean	5.36^a^	5.94	2.66	14.58^a^	13.25	7.24^a^	14.04^a^	12.33^a^	11.91	7.40^a,b^	9.29^a^	14.79^b^	11.03^b^
	Std. Deviation	0.79	1.95	0.88	1.61	1.75	1.99	2.45	1.97	2.30	1.65	2.65	0.37	0.37
	Minimum	3.8	2.3	1.1	11.6	10.5	3.8	10.8	8.8	8.0	4.9	5.5	14.2	10.5
	Maximum	7.7	9.6	4.5	16.9	18.0	11.1	18.2	17.7	17.7	11.8	15.5	15.7	11.7
Pattern III (n=32)	Mean	5.96^b^	6.42	2.30	15.80^b^	13.07	15.42^b^	8.81^b^	13.98^b^	12.83	8.59^b^	10.68^b^	19.19^c^	14.68^c^
	Std. Deviation	1.03	1.77	0.69	3.10	2.67	2.18	4.05	2.78	2.23	2.89	2.63	0.68	0.61
	Minimum	4.3	3.5	0.9	11.2	8.4	11.2	4.6	9.0	8.7	4.4	6.9	18.1	13.2
	Maximum	8.8	10.0	3.0	21.0	18.1	18.8	17.4	17.1	17.1	13.3	16.9	20.4	15.7
F value		4.663	1.290	1.375	3.258	0.810	164.428	23.453	4.988	2.057	5.641	3.763	484.340	225.095
p value		0.012*	0.281	0.259	0.043*	0.449	<0.001*	<0.001*	0.009*	0.134	0.005*	0.027*	<0.001*	<0.001*

^a,b,c^ Different superscripts letters along the columns indicate statistically significant differences determined using Tukey’s *post-hoc* test. F value from ANOVA. *p<.05 indicate statistical significance from ANOVA test.

**Table 4: t4:** Distribution and comparison of facial soft tissue thickness and pharyngeal airway widths in Pattern I, II and III skeletal malocclusion among male participants.

Malocclusion		Measurements												
		GLS-GL	NS-N	RH	SN-A	LS-PR	ST-U1	LI-ID	B-LM	POGS-POG	MES-ME	GNS-GN	Upper airway	Lower airway
Pattern I (n=17)	Mean	6.05	6.71	2.87	16.97	16.40^a^	7.89^a^	13.45^a^	12.77	12.55	8.55	11.44	17.94^a^	13.37^a^
	Std. Deviation	1.23	1.58	0.76	3.25	1.95	2.72	3.56	1.36	2.14	1.77	2.77	0.46	0.92
	Minimum	3.2	3.4	2.0	12.7	13.4	4.0	4.7	10.7	8.3	5.4	6.4	17.2	11.1
	Maximum	7.9	9.5	4.6	23.7	20.8	15.4	19.3	15.5	17.7	12.1	18.0	18.7	14.9
Pattern II (n=14)	Mean	5.75	7.16	3.21	16.84	15.58^a^	8.30a	15.27^b^	12.96	13.34	8.56	11.14	14.67^b^	10.92^b^
	Std. Deviation	0.99	1.60	0.91	1.93	1.71	1.88	2.70	1.97	1.84	1.54	2.62	0.42	0.46
	Minimum	4.2	4.4	2.1	12.9	11.9	5.5	10.6	8.6	10.3	6.3	7.0	14.2	9.7
	Maximum	7.7	10.0	5.3	19.7	18.6	11.5	18.9	17.0	17.6	12.3	16.0	15.7	11.7
Pattern III (n=11)	Mean	5.78	6.82	2.51	16.07	12.41^b^	15.39^b^	11.14^a,b^	14.36	12.24	7.36	9.63	19.29^c^	15.04^c^
	Std. Deviation	0.72	2.07	0.40	2.89	3.25	1.95	4.90	2.56	2.41	1.63	2.08	0.63	0.53
	Minimum	4.3	3.5	1.8	12.3	8.4	13.4	4.6	10.4	8.7	4.4	6.9	18.3	14.2
	Maximum	7.2	10.1	2.9	20.3	18.1	18.7	16.2	17.1	17.1	9.8	12.2	20.5	15.8
F value		0.378	0.268	2.675	0.378	10.665	41.948	3.830	2.483	0.945	2.127	1.801	296.654	111.045
p value		0.688	0.766	0.082	0.688	<0.001*	<0.001*	0.030*	0.097	0.397	0.133	0.179	<0.001*	<0.001*

^a,b,c^ Different superscripts letters along the columns indicate statistically significant differences determined using Tukey’s *post-hoc* test. F value from ANOVA. *p<.05 indicate statistical significance from ANOVA test.

**Table 5: t5:** Gender-wise distribution and comparison of facial soft tissue thickness and pharyngeal airway widths among participants with Pattern I skeletal malocclusion.

Sex		Measurements												
		GLS-GL	NS-N	RH	SN-A	LS-PR	ST-U1	LI-ID	B-LM	POGS-POG	MES-ME	GNS-GN	Upper airway	Lower airway
Female (n=26)	Mean	5.44	5.67	2.53	14.35	13.84	7.15	12.47	12.32	11.86	6.79	9.19	17.89	13.24
	Std. Deviation	0.58	1.73	1.02	2.02	2.554	1.90	2.14	2.18	1.56	1.12	1.54	0.57	0.95
	Minimum	4.3	2.4	1.1	11.2	9.4	3.7	9.6	8.9	7.7	5.1	6.8	17.1	11.7
	Maximum	6.9	8.6	6.3	19.4	20.0	10.8	16.3	17.8	15.5	9.5	12.5	18.7	15.0
Male (n=17)	Mean	6.05	6.71	2.87	16.97	16.40	7.89	13.45	12.77	12.55	8.55	11.44	17.94	13.37
	Std. Deviation	1.23	1.58	0.76	3.25	1.95	2.72	3.56	1.36	2.14	1.77	2.77	0.46	0.92
	Minimum	3.2	3.4	2.0	12.7	13.4	4.0	4.7	10.7	8.3	5.4	6.4	17.2	11.1
	Maximum	7.9	9.5	4.6	23.7	20.8	15.4	19.3	15.5	17.7	12.1	18.0	18.7	14.9
t value		-2.191	-2.008	-1.188	-3.277	-3.518	-1.063	-1.140	-0.754	-1.215	-4.003	-3.434	-0.295	-0.444
p value		0.0034*	0.051	0.242	0.002*	0.001*	0.294	0.261	0.455	0.231	<0.001*	0.001*	0.769	0.660

t value: from independent sample t test. *p<.05 indicate statistical significance.

**Table 6: t6:** Gender-wise distribution and comparison of facial soft tissue thickness and pharyngeal airway widths among participants with Pattern II skeletal malocclusion

Sex		Measurements												
		GLS-GL	NS-N	RH	SN-A	LS-PR	ST-U1	LI-ID	B-LM	POGS-POG	MES-ME	GNS-GN	Upper airway	Lower airway
Female (n=29)	Mean	5.36	5.94	2.66	14.58	13.25	7.24	14.04	12.33	11.91	7.40	9.29	14.79	11.028
	Std. Deviation	0.79	1.95	0.88	1.61	1.76	1.99	2.45	1.97	2.30	1.65	2.65	0.37	.3673
	Minimum	3.8	2.3	1.1	11.6	10.5	3.8	10.8	8.8	8.0	4.9	5.5	14.2	10.5
	Maximum	7.7	9.6	4.5	16.9	18.0	11.1	18.2	17.7	17.7	11.8	15.5	15.7	11.7
Male (n=14)	Mean	5.75	7.16	3.207	16.84	15.58	8.30	15.27	12.96	13.34	8.56	11.14	14.67	10.921
	Std. Deviation	0.99	1.60	0.91	1.93	1.70	1.88	2.70	1.97	1.84	1.54	2.62	0.42	.4577
	Minimum	4.2	4.4	2.1	12.9	11.9	5.5	10.6	8.6	10.3	6.3	7.0	14.2	9.7
	Maximum	7.7	10.0	5.3	19.7	18.6	11.5	18.9	17.0	17.6	12.3	16.0	15.7	11.7
t value		-1.385	-2.027	-1.890	-4.026	-4.118	-1.661	-1.496	-0.995	-2.032	-2.200	-2.154	0.966	0.819
p value		0.173	0.049*	0.066	<0.001*	<0.001*	0.104	0.142	0.326	0.049*	0.033*	0.037*	0.340	0.341

t value: from independent sample t test; *p<.05 indicate statistical significance.

**Table 7:** Gender-wise distribution and comparison of facial soft tissue thickness and pharyngeal airway widths among participants with Pattern III skeletal malocclusion.

Sex		Measurements												
		GLS-GL	NS-N	RH	SN-A	LS-PR	ST-U1	LI-ID	B-LM	POGS-POG	MES-ME	GNS-GN	Upper airway	Lower airway
Female (n=32)	Mean	5.96	6.42	2.30	15.80	13.07	15.42	8.81	13.98	12.83	8.59	10.68	19.19	14.68
	Std. Deviation	1.03	1.77	0.69	3.10	2.67	2.19	4.05	2.78	2.23	2.89	2.63	0.68	0.61
	Minimum	4.3	3.5	.9	11.2	8.4	11.2	4.6	9.0	8.7	4.4	6.9	18.1	13.2
	Maximum	8.8	10.0	3.0	21.0	18.1	18.8	17.4	17.1	17.1	13.3	16.9	20.4	15.7
Male (n=11)	Mean	5.78	6.82	2.52	16.07	12.41	15.39	11.14	14.36	12.24	7.36	9.63	19.29	15.04
	Std. Deviation	0.72	2.07	0.40	2.89	3.25	1.95	4.90	2.56	2.41	1.63	2.08	0.63	0.53
	Minimum	4.3	3.5	1.8	12.3	8.4	13.4	4.6	10.4	8.7	4.4	6.9	18.3	14.2
	Maximum	7.2	10.1	2.9	20.3	18.1	18.7	16.2	17.1	17.1	9.8	12.2	20.5	15.8
t value		0.537	-0.619	-0.984	-0.253	0.670	0.033	-1.557	-0.392	0.740	1.341	1.201	-0.414	-1.749
p value		0.594	0.539	0.331	0.802	0.507	0.974	0.127	0.697	0.464	0.187	0.237	0.681	0.088

t value: from independent sample t test; *p<.05 indicate statistical significance.

### SAMPLE SIZE DETERMINATION

n = sample size in each group

µ_1_ = population mean in treatment Group I

µ_2_ = population mean in treatment Group 2

µ_1_- µ_2_= the difference the investigator wishes to detect

σ^2^= population variance (SD)

a = conventional multiplier for alpha= 0.05

b = conventional multiplier for power= 0.80

$$n=\frac{2\left[{\left(a+b\right)}^{2}\sigma^{2}\right]}{{\left(\mu_{1}-\mu_{2}\right)}^{2}}$$

Considering the parameters of mean and standard deviation of upper and lower pharyngeal airway space in Pattern I, Pattern II, Pattern III malocclusions from a previous study^20^, 95% confidence interval, 80% power and a sample ratio of 1:1, the sample size was estimated to include 43 in each group. According to facial soft tissue data obtained from previous research, a minimum of 21 patients were required to participate in each group. Therefore, a maximum sample size of 43 was considered in each group

## STATISTICAL ANALYSIS

Statistical analysis was performed using SPSS. The reliability of measurements of airway space and soft tissues was examined by calculating intraclass correlation values. One-way ANOVA was used to compare the mean values of measurements of facial soft tissue thickness and airway space of the three groups, along with Tukey *post-hoc* test. P<0.05 was considered statistically significant.

## RESULTS

There was a statistically significant difference in facial soft tissue thickness in the different skeletal patterns. *Labrale superius* and stomion were significantly higher in skeletal Pattern III and smaller in Pattern II. *Labrale inferius* was found to be significantly higher in skeletal Pattern II and smaller in Pattern III. Though not statistically significant, the thickness at menton, pogonion and gnathion was higher in Pattern II, average in Pattern I and smaller in Pattern III. The increased soft tissue thickness in menton, pogonion, gnathion in skeletal Pattern II might be due to a soft tissue compensation for deficient mandible, and decreased soft tissue thickness in skeletal Pattern III mandible might be due to a prognathic mandible. Sexual dimorphism was noted. Men had a greater soft tissue thickness than women in all sites. Both upper and lower pharyngeal airway widths were found to be significantly higher in skeletal Pattern III and smaller in skeletal Pattern II.

## DISCUSSION

The facial profile reflects the variability of soft tissue thickness and should be a factor in the diagnosis and the planning of orthodontic treatment.^11^ Evaluation of the soft tissues plays an important role in diagnosis, treatment planning and obtaining facial harmony.^.^ Arnett and Gunson^13^ suggested that the patient should be positioned in a relaxed lip position while evaluating the soft tissue profile, since this position demonstrates the relationship of soft tissues to hard tissues without muscular compensation for dentoskeletal abnormalities. According to Uysal et al.,^14^ the relaxed lip position was also used for standardization of the method, when taking the cephalograms, for accurate assessment of the soft tissues. In agreement with different studies,^15^ ^,^ ^16^ the relaxed lip position was used in the present study when taking the cephalograms, in order to ensure accurate assessment of soft tissue thickness. As has been shown in numerous studies, mean age of the subjects examined might also affect the soft tissue thickness.^17^ Subjects selected in this study were of the age group 16 -31 years, where no much changes occur, as the soft tissue thickness will have reached adult size, according to Subtenly.^18^ A history of trauma, fall, surgery of nose, any nasal obstruction was assessed, and if present, patients were excluded.

A study by Naoumova et al.^19^ compared the accuracy of cephalometric measurements made with digital tracing software (Facad^®^) and equivalent hand-traced measurements, to evaluate the reproducibility of each method; and, according to this study, Facad^®^ is reliable and can be used routinely. 

Hence, in this study Facad^®^ cephalometric software was used to compare the facial soft tissue thickness and pharyngeal airway widths in the different skeletal patterns. 

There was statistically significant difference in facial soft tissue thickness between the different skeletal patterns. Notable difference was identified in *labrale superius*, *labrale inferius* and stomion. *Labrale superius* was significantly higher in skeletal Pattern III (14.85± 2.6318mm) and smaller in skeletal Pattern II (12.9±2.8mm, p value -0.02). This finding might be due to the angulation of the maxillary and mandibular central incisors. According to Kamak et al^20^, this might be due to inclination of incisors. The maxillary incisors are tipped labially and the mandibular incisors, lingually in patients with Class III malocclusion. Mandibular anterior teeth might push the upper lip upward and outward. This result correlates with the study conducted by Chaudhary et al. ^21^, who found that the upper lip thickness is higher for Class III (13.26 ± 2.32mm) and lowest for Class II (12.67 ± 2.69mm). They reasoned that, since most of the skeletal Class III malocclusion has maxillary hypoplasia, there is an increase in soft tissue volume to mask the degree of hypoplasia, resulting in a thick upper lip. This might be the reason for increased upper lip thickness in skeletal Class III malocclusion. This is in agreement with study by Yan et al.^22^, which found an increased upper lip thickness in skeletal Class III malocclusion. According to Thuer and Ingervall’s^23^ findings, the labial pressure from upper lip in Class III malocclusion was very low, due to the retrognathic maxilla, thus resulting in reduced lip strain and increased thickness. In other words, the retrognathic maxillary position can be related to the lower circumoral pressure. All these findings support the finding of significantly higher values of *Labrale superius* in skeletal Class III malocclusion and smaller in skeletal Class II, and explain why stomion and *Labrale inferius* were found to be statistically significant and higher in skeletal Class III (15.41± 2.1mm) malocclusion, and smaller in skeletal Class II (7.59±2mm, p value <0.01). In the present study, sexual dimorphism was noted: men had a greater soft tissue thickness than women in all sites. However, statistically significant gender differences were not determined for all of the points in each skeletal pattern. 

Though not statistically significant, the thickness at menton, pogonion and gnathion was higher in Pattern II, average in Pattern I and smaller in Pattern III. The increased soft tissue thickness in menton, pogonion and gnathion in Pattern II might be due to a soft tissue compensation for deficient mandible; and decreased soft tissue thickness in Pattern III might be due to a prognathic mandible. In the study conducted by Tiwari et al.^24^, a significant soft-tissue chin thickness was observed in skeletal Class I and Class III malocclusion. This finding is in agreement with the study conducted by Jabbar et al,^10^ who reported that a significant soft tissue chin thickness difference among different skeletal malocclusions was observed. Often a severe skeletal problem is masked by favorable soft tissues. 

The upper airway is a complex structure whose functions consist of respiration, deglutition and phonation. Lower airways includes trachea, bronchi and bronchioles. The bidirectional relationship between breathing and facial growth has been extensively demonstrated: the impact of the breathing pattern and head posture on the facial growth pattern was described in the soft-tissue stretching hypothesis by Solow and Kreiborg.^25^ ^,^ ^26^ There is a close relationship between the pharynx and the dentofacial structures, thus a mutual interaction is expected to occur between the pharyngeal structures and the dentofacial pattern, justifying orthodontic interest. King^27^ and Tourné^28^ stated that nasopharyngeal depth is established at the early ages of life, after which it usually remains the same. It has been stated, however, that width and height increment of the nasopharynx continues until adulthood and thus the nasopharyngeal area also increases.

Furthermore, it has been reported that the nasopharyngeal airway area increases rapidly until 13 years of age, and after this period the growth slows down.^29^ ^,^ ^30^ Therefore, to rule out the influence of growth, the sample used in this study consisted of subjects from 16 to 30 years of age, in which the sagittal measurements are more stable and have minimal changes with growth and aging process. In the present study, two linear measurements were taken to compare the pharyngeal airway widths among different skeletal patterns. And it was found that there was statistically significant difference in both upper and lower airways. Both the values were higher in Pattern III (19.22±0.67mm, 14.77±0.61mm, p value<0.01) and smaller in Pattern II (14.75±0.39mm, 10.99±0.40mm, p value<0.01). 

Staley et al.^31^ and Lee et al.^32^ hypothesized that a reduction of the airway in patients with skeletal Class II may result from mandibular and tongue posteroinferior movements. This possibility may explain the differences in pharyngeal airway between subjects with skeletal Pattern II and those with skeletal Pattern III in the in the present study. Chokotiya et al.^33^ evaluated the upper and lower pharyngeal airway widths that were affected by different skeletal malocclusions, and found that they were not affected by the changes in the ANB angle, and no significant difference was found between the sexes. 

Obstructive sleep apnea syndrome is characterized by temporary occlusion of the upper airway several times during the night, which may result in hypoxia and sleep fragmentation, with symptoms as chronic tiredness, day-time somnolence associated with snoring and intellectual deterioration. Hence soft tissue thickness and pharyngeal airway widths must be considered as an important criteria while formulating orthodontic/orthognathic treatment strategies. The results of the present study suggests a strong relationship between facial soft tissue thickness, pharyngeal airway widths and different skeletal patterns.

## CONCLUSION

When developing orthodontic treatment plans, soft tissue differences and pharyngeal airway widths need to be taken into account as crucial factors. The findings of the present study indicate that there is a substantial correlation between the thickness of the soft tissues in the face, the width of the pharynx, and different skeletal patterns. 
